# Assessment of Corneal Biomechanical Properties and Intraocular Pressure in Myopic Spanish Healthy Population

**DOI:** 10.1155/2014/905129

**Published:** 2014-02-25

**Authors:** María A. del Buey, Laura Lavilla, Francisco J. Ascaso, Elena Lanchares, Valentín Huerva, José A. Cristóbal

**Affiliations:** ^1^Department of Ophthalmology, Lozano Blesa University Clinic Hospital, Zaragoza, Spain; ^2^Department of Ophthalmology, Quirón University Hospital, Zaragoza, Spain; ^3^Instituto Aragonés de Ciencias de la Salud (IACS), Zaragoza, Spain; ^4^Aragón Institute for Engineering Research (I3A), University of Zaragoza, CIBER on Bioengineering, Biomaterials and Nanomedicine (CIBER-BBN), Zaragoza, Spain; ^5^Department of Ophthalmology, Arnau de Vilanova University Hospital and IRB-Lleida, Avenida Rovira Roure 80, Lleida, Spain

## Abstract

*Purpose*. To examine biomechanical parameters of the cornea in myopic eyes and their relationship with the degree of myopia in a western healthy population. *Methods*. Corneal hysteresis (CH), corneal resistance factor (CRF), Goldmann correlated intraocular pressure (IOP), and corneal compensated IOP (IOPcc) were measured using the ocular response analyzer (ORA) in 312 eyes of 177 Spanish subjects aged between 20 and 56 years. Refraction was expressed as spherical equivalent (SE), which ranged from 0 to −16.50 diopters (D) (mean: −3.88 ± 2.90 D). Subjects were divided into four groups according to their refractive status: group 1 or control group: emmetropia (−0.50 ≤ SE < 0.50); group 2: low myopia (−0.75 ≤ SE < 3.00 D); group 3: moderate myopia (−3.00 ≤ SE ≤ −6.00 D); and group 3: high myopia (SE greater than −6.00 D). We analyzed the relationship between corneal biomechanics measured with ORA and SE. *Results*. CH in the emmetropia, low myopia, moderate myopia, and high myopia groups was 11.13 ± 0.98, 11.49 ± 1.25, 10.52 ± 1.54, and 10.35 ± 1.33 mmHg, respectively. CH in the highly myopic group was significantly lower than that in the emmetropic group (*P* = 0.07) and low myopic group (*P* = 0.035); however, there were no differences with the moderate myopic group (*P* = 0.872). There were no statistically significant differences regarding IOP among the four groups (*P* > 0.05); nevertheless, IOPcc was significantly higher in the moderately myopic (15.47 ± 2.47 mmHg) and highly myopic (16.14 ± 2.59 mmHg) groups than in the emmetropia (15.15 ± 2.06 mmHg) and low myopia groups (14.53 ± 2.37 mmHg). No correlation between age and the measured parameters was found. CH and IOPcc were weakly but significantly correlated with SE (*r* = 0.171, *P* = 0.002 and *r* = −0.131, *P* = 0.021, resp.). *Conclusions*. Present study showed only a very weak, but significant, correlation between CH and refractive error, with CH being lower in both moderately and highly myopic eyes than that in the emmetropic and low myopic eyes. These changes in biomechanical properties of the cornea may have an impact on IOP measurement, increasing the risk of glaucoma.

## 1. Introduction

Myopia is the most common ocular disorder. Its worldwide prevalence is about 30% and up to 80% in certain Asian populations [1–4]. Corneal hysteresis (CH) is a parameter which measures the viscoelastic behaviour of the cornea, indicating its biomechanical integrity [5]. Some clinical conditions such as keratoconus, Fuchs corneal dystrophy, glaucoma, and corneal refractive surgery may induce changes in corneal biomechanical properties, leading to a decrease in CH [6–10]. Although several studies with the ocular response analyzer (ORA, Reichert Inc., NY, USA) have reported a relationship between the refractive error and corneal biomechanical properties, it is still under debate [4, 11]. Thus, whereas in several studies CH was found significantly lower in patients with high myopia [12–17], other authors did not find any correlation [18–20]. Most of the studies were performed in myopic Singaporean and Chinese populations [12, 14, 18, 20] and others in Brazilian [21] or Turkish people [15], with only a few ones in Caucasian individuals [13, 16]. Moreover, since biomechanical properties of the cornea are known to change with age [22], some slightly mixed findings in children may not be applicable to adult populations [16]. The aim of present study was to measure with the ORA device several corneal biomechanical parameters in an adult western healthy population containing emmetropes, low myopes, moderate myopes and highly myopic individuals, and the relationship between these parameters and the values of intraocular pressure (IOP) determined with ORA, including Goldmann correlated IOP (IOP) and corneal compensated IOP (IOPcc).

## 2. Methods

In this observational comparative study, 312 eyes of 177 healthy subjects were analyzed (76 men and 101 women). They were recruited sequentially among patients and healthy volunteers in the Department of Ophthalmology at the Lozano Blesa University Clinic Hospital and Quirón University Hospital, Zaragoza, Spain. The average age of the patients was 33.27 ± 7.65 years (range, 20–56). All subjects received a complete ophthalmic examination including measurement of best-corrected visual acuity (BCVA) with ETDRS chart, slit-lamp anterior segment biomicroscopy, fundus examination, and corneal topography (Orbscan II) in order to discard the existence of subclinical corneal pathology. Automated and subjective refractions were performed to determine refractive error in order to use it for the statistical analyses. Their spherical equivalent (SE) of refractive error ranged continuously from 0 to −16.50 D (mean: −3.88 ± 2.90 D). For the purpose of the study, subjects were divided into four groups according to their refractive status: group 1 or control group: emmetropia (−0.50 ≤ SE < 0.50); group 2: low myopia (−0.75 ≤ SE < −3.00 D); group 3: moderate myopia (−3.00 ≤ SE ≤ −6.00 D); and group 4: high myopia (SE greater than −6.00 D). All participants had monocular BCVA of 20/32 (0.20 logMar notation) or better. Subjects who had refractive errors such as hyperopia > 0.5 D or astigmatism > 1 D, IOP > 21 mmHg, signs of glaucomatous optic neuropathy, family history of glaucoma in a first-degree relatives, corneal dystrophy, and myopic macular degeneration, those who had undergone previous eye surgery or trauma, eye infection, diabetes mellitus, corticosteroid use or other acute or chronic diseases, or using any topical eye medication, or subjects that did not meet normal topographic criteria were excluded from the study.

Corneal biomechanical properties, such as CH and CRF, were measured by the same masked technician with the Ocular Response Analyzer (ORA software version 2.04; Reichert Ophthalmic Instruments, Buffalo, NY) using standard technique [5, 8, 23]. Briefly, a rapid air puff deformed the cornea, and the induced corneal deformation was detected with an electrooptical system. The air pulse induced inward, and then outward, corneal movement, which provided two applanation measurements. CH resulted from the damping of the cornea because of its biomechanical properties and was derived from the difference of the two measurements during the applanation process. CRF, also derived from corneal hysteresis, is calculated as a linear function of the pressures corresponding to the two applanations. CRF is an indicator of the overall resistance of the cornea, which, according to previous data, seems to be related to central corneal thickness (CCT) and GAT determined IOP, but not to IOPcc [5]. The ORA also determined the values of noncontact tonometer Goldmann correlated IOP and IOPcc. IOPcc is a pressure measurement that utilizes the new information provided by the CH measurement to provide an IOP measurement less affected by corneal properties. CCT was measured, following corneal biomechanical properties measurements, by ultrasound pachymetry (20 MHz) using an ORA-integrated hand-held pachymeter. Three measurements of good quality were obtained for each patient; the signals with the highest Waveform Score (WS) were highlighted as the best score value (BSV) and were used for statistical evaluation. The study and data accumulation were performed with the approval of the local ethics committee, informed consent was obtained from each subject participating in the study, and the study protocol was consistent with the tenets of the Declaration of Helsinki.

## 3. Data Analysis

Values were presented as mean ± SD. Statistical analyses were conducted using a commercial software (SPSS software, version 13.0; SPSS, Inc., Chicago, IL). The distribution of the measured variables was estimated by the one-sample Kolmogorov-Smirnov test. One-way analysis of variance (ANOVA) and post hoc tests were used for determining whether the values of a particular variable differed between the three diagnostic groups. We combined data from both eyes using the mixed model method [24], which adjusts for the correlation between the two eyes in a unique person. Multivariate mixed-model analysis adjusted by age and sex was used to determine the relationship between two continuous variables. Pearson's correlation coefficient (*r*) was used to assess the relationship between spherical equivalent power and the corneal biomechanical properties (CH and CRF) as well as IOP (IOPcc and IOP) and CCT. The level of statistical significance was set to *P* < 0.05.

## 4. Results

**Figure 1:**
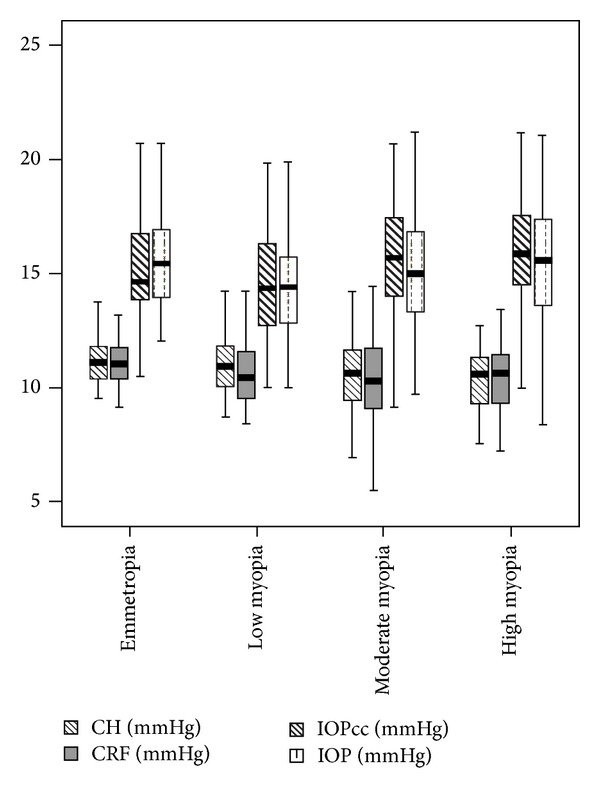
Box-and-whisker plot for corneal hysteresis (CH) and corneal resistance factor (CRF) and noncontact tonometer intraocular pressure (IOP) and corneal compensated intraocular pressure (IOPcc). Average and standard deviation values are presented in mmHg.

A total of 177 patients (312 eyes) were enrolled in this study. The refraction among all included eyes ranged from 0 to −16.50 D (SE). 45 eyes corresponding to 25 patients (15 women and 10 men) were included as healthy controls (group 1). 71 eyes of 47 patients (20 women and 27 men) were included in group 2. One hundred forty-five eyes of 72 patients were included in group 3. And, finally, 51 eyes of 33 patients (23 women and 10 men) were included in group 4. Significant differences in refraction distribution were found between the four diagnostic groups (*P* < 0.05; Table 1). The one-sample Kolmogorov-Smirnov test showed that CH, CRF, IOP, IOPcc, and CCT were normally distributed (*P* = 0.77, 0.78, 0.92, 0.42, and 0.51, resp.) (Figure 1). Parameters such as age, BCVA, IOP, and CCT were not significantly different among groups (*P* > 0.05) (Table 2). CH in the emmetropia, low myopia, moderate myopia, and highly myopic groups were 11.08 ± 0.98, 11.00 ± 1.25, 10.52 ± 1.54 mmHg, and 10.35 ± 1.33, respectively (Table 2). CH in the moderately and highly myopic groups was significantly lower than in the emmetropic (*P* = 0.02 and *P* = 0.01, resp.; Games-Howell post hoc test) and low myopic group (*P* = 0.07 and *P* = 0.04, resp.; Games-Howell post hoc test). CRF of the emmetropic group was significantly higher than that in the moderately and highly myopic groups (*P* < 0.001 and *P* = 0.04, resp.; Games-Howell post hoc test). There was no significant difference in IOP (*P* = 0.083 ANOVA test; *P* > 0.05 least significant difference post hoc test); however, IOPcc was significantly higher in the moderate (15.47 ± 2.47) and highly myopic (16.14 ± 2.59) groups compared to the emmetropia group (15.15 ± 2.06) (*P* = 0.046 resp. to highly myopic group; least significant difference post hoc test) and low myopia group (14.53 ± 2.37) (*P* = 0.008 and *P* < 0.001, resp.; least significant difference post hoc test) (Table 2).

**Figure 2:**
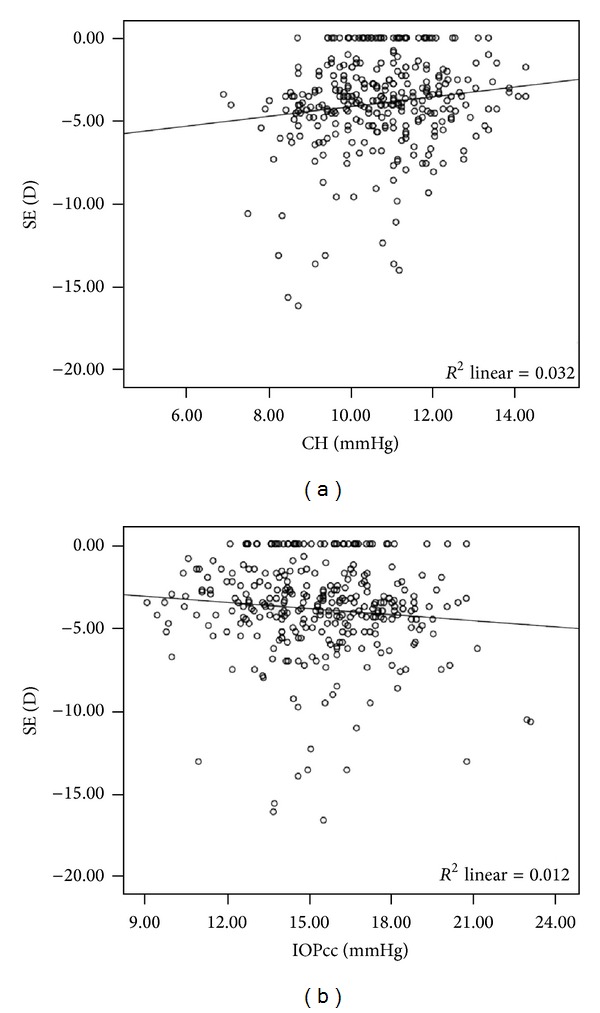
Correlation between spherical equivalent (SE) and (a) corneal hysteresis (CH) and (b) corneal compensated intraocular pressure (IOPcc).

No correlations were found in the measured parameters with the age. CH and IOPcc were parameters significantly correlated with SE (*r* = 0.171,  *P* = 0.013 and *r* = −0.131, *P* = 0.021, resp., Pearson's correlation coefficient) (Figure 2).

## 5. Discussion

Corneal hysteresis is the result of viscoelastic properties of the cornea, together with the combined effect of the corneal thickness and rigidity [5]. Low values of CH indicate a soft or floppy cornea. According to Reichert, CRF is dominated by the corneal elastic properties and appears to be an indicator of the overall resistance of the cornea [5, 23, 25]. There is strong evidence that corneal biomechanical properties are correlated to the degree of myopia. Thus, the abnormal elongation of the myopic eye is associated with corneal flattening and thinning [11], resulting in a decreased CH and CRF [12, 26]. Moreover, myopic eyes have a lower ocular rigidity than emmetropic and hyperopic eyes [27, 28]. Therefore, corneal rigidity, as part of the global rigidity, is likely to be low in myopic eyes as suggested by the decreased CH. Finally, corneal biomechanical properties reflect viscoelastic characteristics of the cornea and the mechanical strength of stromal collagen fibrils interacting with the extracellular proteoglycan matrix [22].

Several studies have used ORA to study the correlation between corneal biomechanics and the degree of myopia. Nevertheless, the results are contradictory. Thus, whereas some authors found a significantly lower CH in highly myopic patients compared with nonmyopic or low myopia subjects [12–17], other studies did not show a correlation between CH and myopia [18–20]. This could maybe be due to the narrow range of myopia selected for the individuals [20]. We found that CH in moderate and highly myopic groups was significantly lower than that in the emmetropic/low myopic group. However, our study differs in a few aspects. Most of the studies were performed in myopic Asian populations, whereas we measured corneal biomechanical characteristics in a western population. Furthermore, we analyzed these parameters in adults, avoiding the possible influence of age if children would have been included. We included not only highly myopic eyes but also moderate myopes and emmetropes/low myopes. Finally, an exhaustive selection of the subjects was carried out for the present study, excluding eyes with topographical patterns suspected or indicatives of keratoconus as well as signs of endothelial damage or glaucoma, since these disorders would cause a decrease in CH.

It has been suggested that the elastic properties of the cornea may influence the accuracy of IOP measurement [22]. In that case, the ORA may be useful for the diagnosis and management of glaucoma and ocular hypertension (OHT) [25]. Several studies have reported that myopic subjects, especially in the highly myopic group, showed higher IOP than controls [12, 29–32]. Altan et al. [15] found that IOPcc measured by ORA, but not IOP, was significantly higher in highly myopic eyes than in nonmyopia or low myopia group. Additionally, we observed that IOPcc was significantly higher in both the moderately and highly myopic groups compared to the emmetropia/low myopia group. Several studies [15, 20] have revealed a significant correlation between axial length and IOPcc. The positive correlations between CCT and CH have also been confirmed [5, 23, 33]. In accordance with Altan et al. [15], IOP and IOPcc were also significantly correlated with CCT. However, we found a significant relationship between IOP and IOPcc with CH, but not with CCT. The changes in CH with refraction are not related to the differences in CCT [16]. CCT in all our myopic patients was typically within normal limits and independent of the degree of myopia, in agreement with previous studies which found no significant differences between myopes and emmetropes in terms of corneal thickness [34, 35].

Unlike previous studies, the present study showed that not only high myopic eyes but also moderately myopic ones have a compromised corneal mechanical strength. These inconsistent results could be justified by differences related to other factors such as race, range of age, gender, refractive status, axial length, corneal curvature, and CCT. [14, 19, 20] Although there is a higher prevalence of glaucoma among myopic eyes than that in nonmyopic eyes [29, 32], it is still unclear why myopia increases IOP. Jiang et al. [14] attributed these differences of corneal biomechanical properties to the difference of age between groups. Nevertheless, in our study the variable age was not significantly different among the four groups.

The main limitations of this study would include that axial length and corneal curvature were not measured. Because of this, the importance of both factors in the refraction-related mechanical changes of the cornea is unknown.

Concluding, the present study shows a very weak but significant correlation between CH and refractive error, with CH being lower in both moderately and highly myopic groups than that in the emmetropic/low myopic ones, indicating that some aspects of corneal biomechanics may need to be interpreted in light of the refraction, especially in myopia. These changes in biomechanical properties of the cornea may have an impact on IOP measurement, increasing the risk of glaucoma. Further studies with larger sample size should be performed.

## Figures and Tables

**Table 1:** Baseline data of the four diagnostic groups.

Parameters	Emmetropia (45 eyes/25 patients)	Low myopia (71 eyes/47 patients)	Moderate myopia (145 eyes/72 patients)	High myopia (51 eyes/33 patients)	*P*
SE (D)	0.25 ± 0.43	−2.15 ± 0.69	−4.23 ± 0.77	−8.69 ± 2.88	<0.001^a^
Sex (M/F)	10/15	27/20	29/43	10/23	0.057^b^
Age (years)	35.37 ± 7.73	33.60 ± 7.12	32.24 ± 6.68	33.88 ± 9.85	0.091^a^

D: diopters; F: female; M: male.

Data were presented as mean ± SD of the indicated variables.

^a^One-way analysis of variance.

^b^ *χ*-test.

Significant differences in refraction were present among the four groups (*post hoc* test, *P* < 0.05).

**Table 2:** Differences in corneal biomechanical parameters and CCT in the four diagnostic groups.

Parameters	Emmetropia (45 eyes/25 patients)	Low myopia (71 eyes/47 patients)	Moderate myopia (144 eyes/72 patients)	High myopia (51 eyes/33 patients)
CH (mmHg)	11.08 ± 0.98 (9.51 ± 13.70)	11 ± 1.25 (8.70–14.20)	10.52 ± 1.54 (5.02–14.20)	10.35 ± 1.33 (7.48–12.7)
CRF (mmHg)	11.07 ± 1.06 (9.15 ± 13.70)	10.63 ± 1.39 (8.40–14.20)	10.34 ± 1.64 (5.46–14.40)	10.36 ± 1.46 (7.23–13.40)
CCT (*μ*m)	573.82 ± 38.03 (513–653)	557.29 ± 38.03 (500–658)	553.22 ± 34.21 (466–658)	552.79 ± 26.86 (463–595)
IOP (mmHg)	15.61 ± 2.23 (11.96–20.7)	14.55 ± 2.52 (10–20.2)	15.05 ± 2.53 (9.7–21.2)	15.54 ± 2.78 (8.36–21.06)
IOPcc (mmHg)	15.15 ± 2.06 (10–18.50)	14.53 ± 2.37 (10–19.80)	15.47 ± 2.47 (9.1–20.7)	16.14 ± 2.59 (10–23)

CH: corneal hysteresis; CRF: corneal resistance factor; CCT: central corneal thickness; IOP: noncontact tonometer intraocular pressure; IOPcc: corneal compensated IOP.

Data were presented as mean ± SD of the indicated variables.
